# Opto-Electrochemical Probes for In Vitro/In Vivo Analysis: Principles, Designs, and Applications

**DOI:** 10.3390/bios16060319

**Published:** 2026-06-02

**Authors:** Alexander N. Vaneev, Petr V. Gorelkin, Natalia L. Klyachko, Alexander S. Erofeev

**Affiliations:** 1 Research Laboratory of Biophysics, National University of Science and Technology “MISIS”, 119049 Moscow, Russia; 2 Chemistry Department, Lomonosov Moscow State University, 119991 Moscow, Russia

**Keywords:** nanoelectrodes, optical fiber, single-cell analysis, scanning probe microscopy, nanoendoscopy, electrochemical sensing, scanning ion-conductance microscopy

## Abstract

This review examines recent advances in multifunctional probes that integrate optical and electrochemical channels for in vitro/in vivo studies. Integration of electrodes with optical fibers provides a powerful platform for localized light delivery and simultaneous electrochemical detection of cellular metabolites both within and at the surface of single living cells. These hybrid devices bridge optical stimulation methods, including optogenetics, and electrochemical monitoring of the cellular response within the same experimental preparation. The review systematically categorizes distinct probe architectures: optical nanoendoscopes for intracellular measurements, probes with a shared opto-electrochemical channel, devices where optical and electrochemical channels are physically separated, and probes engineered for neural interfaces and scanning probe microscopy. For each category, fabrication approaches, surface modification strategies, and representative biological applications are discussed. Particular attention is given to the fundamental tension between optical transparency and electrical conductivity in shared-channel designs, to the mechanical requirements imposed by neural tissue on implantable probes, and to the spatial resolution limits of current scanning probe platforms. The review concludes with a critical assessment of current limitations and future directions, including higher spatial resolution, simultaneous multiplexed analyte detection and broader translation of these technologies toward in vivo experimental models.

## 1. Introduction

Nanoscale devices for single-cell investigation have attracted considerable attention over the past decade due to their capacity for quantitative measurements with nanometer-scale resolution [1,2,3,4,5,6]. Understanding molecular processes within living cells remains one of the central challenges of modern biomedicine [7]. However, traditional imaging methods have significant limitations. Electron microscopy requires sample fixation, which precludes the study of dynamic processes. Fluorescence microscopy records the position of labels rather than the target molecules themselves and does not provide direct measurement of cellular metabolite concentrations [8,9]. These limitations have driven the development of alternative approaches based on probes capable of penetrating cells and performing local measurements in real time [10,11,12,13,14].

Micro- and nanoelectrodes are well established in cell biology, optogenetics and electrochemistry [15,16,17,18]. The reduced double-layer capacitance of nanoelectrodes enables rapid electrochemical response and facilitates the detection of short-lived intermediates, such as reactive oxygen and nitrogen species (ROS/RNS). The small dimensions and conical or cylindrical geometry of these electrodes permit minimally invasive intracellular measurements. Despite advances in electrochemical sensors capable of monitoring key biochemical parameters of single cells (ROS, pH, metal ions) [19,20,21], technologies integrating electrochemical measurements with controlled optical stimulation remain at an early stage of development [22]. Of particular significance are developments in reducing fiber electrode dimensions to the nanometer scale.

A parallel line of development concerns optical fiber nanoendoscopes, which allow high-resolution visualization and interaction with single cells [23,24]. The integration of micro- and nanoelectrodes with optical fibers enables photoelectrochemical reactions and quantitative assessment of various biochemical processes under localized light illumination in small volumes including droplets, microemulsions and even within single living cells [25]. The advantages of fiber optics such as minimal invasiveness, high flexibility, the ability to deliver light directly to the measurement site and compatibility with electrochemical methods have made fiber optics an indispensable tool in cell biology for investigating of intracellular processes in real time without substantial cellular damage. Consequently, optical fibers have emerged as an important complementary tool in electroanalytical chemistry, providing activation of photosensitive processes and visualization at the micro- and nanoscale in biological systems [26].

Such hybrid devices find application across a wide range of fields from fundamental studies of cellular metabolism to the development of methods for evaluating photodynamic therapy efficacy and neural interfaces for optogenetics [27,28]. Integration of optical fibers with advanced technologies such as optogenetics opens new avenues for controlling and studying single cells. For instance, optical neural interfaces combining fiber optic and optogenetic technologies enable precise cell-type-specific optical addressing in vivo with millisecond temporal resolution. Optrodes with integrated electrodes offer a unique advantage, permitting simultaneous optical stimulation and electrical recording from the same neuronal population [29].

**Figure 1:**
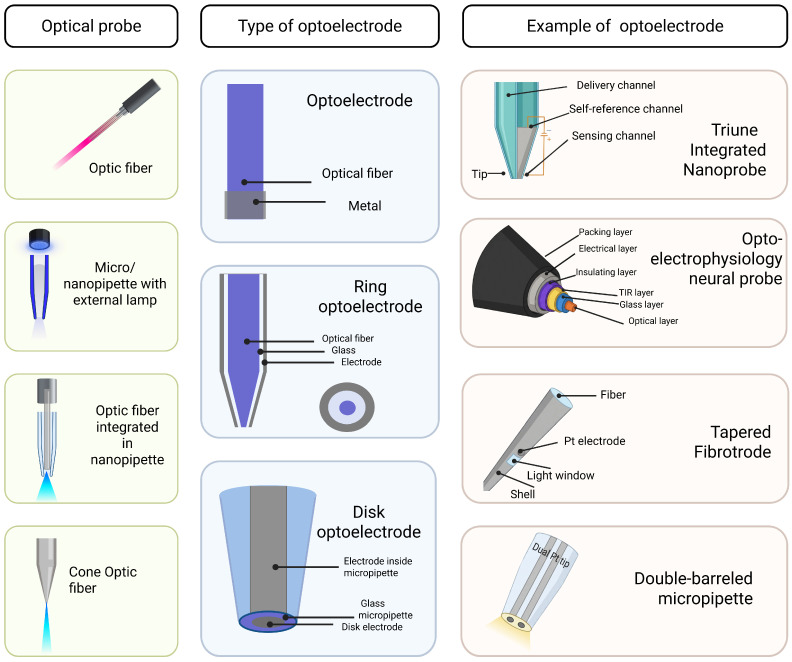
Representative optical probes, optoelectrode configurations, and integrated optoelectrode devices used for simultaneous optical stimulation and electrical/electrochemical recording.

This review systematically examines current methods for probe fabrication, surface modification, and their applications in living cell research, including biosensing. Analytical performance characteristics and applications are discussed across a range of contexts, from intracellular metabolite detection to neurobiology. The central focus is the integration of optical and electrochemical modalities into multifunctional probes capable of addressing key challenges in cell biology and neuroscience (Figure 1).

## 2. Optical Nanoendoscopes for Single-Cell Imaging

High-resolution single-cell visualization is central to numerous scientific disciplines from biology to medicine [30,31]. Nanoscale endoscopes (nanoendoscopes) offer minimally invasive access to the intracellular environment [32]. Depending on the detection principle, nanoendoscopes can be electrochemical, optical, electrophysiological or topographic. Many recent works combine several of these modalities [23]. This section focuses specifically on optical nanoendoscopes, i.e., probes in which light serves as the primary interrogation modality for intracellular imaging and stimulation at the subcellular scale.

At the macroscale, fiber-optic bundles and single multimode fibers are already established tools for clinical endoscopic imaging [33]. Scaling this approach down to the single-cell level requires submicrometer tapered optical fibers that can be incorporated into portable and flexible systems for minimally invasive imaging of opaque biological samples including tissues [34,35,36,37]. The integration of nanophotonic probes into fiber optic imaging systems allows for light manipulation at the nanoscale within living cells [38,39,40,41].

Several platforms have been used to build optical nanoendoscopes: glass nanopipettes, tapered optical fibers, nano- and microwires, and nanotubes [42]. Among these, glass nanopipettes are the most widely accessible. They are pulled from quartz or borosilicate capillaries (inner diameter of tens of micrometers) using a laser puller and can be adapted to a wide range of tasks [23]. A glass nanopipette can serve as a base for a nanowire or nanotube [43]. A common functionalization strategy involves surface modification with nanoparticles for optical measurements [44]. In optical imaging, nanopipettes are used as carriers for quantum dots or plasmonic nanoparticles, which enhance surface-enhanced Raman scattering (SERS) signals and enable highly sensitive analysis of cellular metabolites [45,46,47]. Furthermore, the use of nanopipettes in scanning ion-conductance microscopy (SICM) enables simultaneous topographic imaging of the cell surface and local electrochemical measurements [48,49,50,51].

Etched or drawn single-mode optical fibers represent an alternative platform that provides subcellular resolution without external optics. Nanoendoscopes for optical cell probing have been developed: a nanowire-based endoscope, a photonic crystal-based endoscope, and a SERS-enabled endoscope [43,52,53]. Furthermore, fluorescence combined with nanowire-based endoscopy can be used not only to deliver light into the cell but also to capture the signal from locally excited quantum dots [43]. The advantages of nanowire-based designs include an extremely small excitation volume and minimal perturbation of the cell membrane.

The single-mode fiber-optic nanoendoscope developed by Cheemalapati et al. consisted of a chemically etched fiber with a base diameter of 8 μm, tapered to ~200 nm, enabling simultaneous delivery and collection of light at the subcellular level without the use of external objective optics [42]. The study experimentally demonstrated localized recording of fluorescence spectra from the nuclei of fibroblasts, Hoechst-labeled hepatocytes, and MDA-MB-231 mitochondria with excitation from both an external source and the endoscope itself, as well as in vivo signal collection from YFP-expressing *C. elegans* nematodes. The authors positioned the work as the first application of the nanoendoscope for in vivo imaging.

One example of a nanowire endoscope was the device proposed by Yan et al., which consisted of a one-dimensional SnO_2_ waveguide with a diameter of 100–250 nm, attached to a conically etched single optical fiber with a diameter of 300–500 nm. The small diameter and mechanical flexibility minimized damage to the membrane and cytoskeleton. No necrosis, apoptosis, or significant cytotoxic stress was observed in HeLa cells following nanowire insertion, whereas insertion of a tapered fiber tip alone resulted in cell death in up to 40% of cases [43]. A key capability of this nanoendoscope was the selective visualization of subcellular structures with high spatial resolution. The nanoendoscope successfully resolved two quantum dot clusters separated by only 2 μm within a living cell. In contrast to conventional wide-field epifluorescence microscopy, which excites the entire cell membrane with a high background signal, the nanoendoscope provided targeted, localized illumination. An important result was the demonstration of spatiotemporal delivery of quantum dots to the nucleus or cytoplasm with activation of photocleavable linkers within 1 min of UV irradiation, significantly outperforming passive delivery systems based on carbon nanotubes [43].

Singhal et al. developed a multifunctional cellular endoscope based on carbon nanotubes enabling cell research, fluid transport, and combined optical and electrochemical diagnostics at the organelle level [54,55]. The design consisted of a multiwalled carbon nanotube (50–60 μm long, 50–200 nm outer diameter) attached to the end of a glass micropipette with epoxy resin, with both ends remaining open for fluid transport. Compared with conventional conical glass pipettes, the cylindrical nanotube geometry allowed deeper cell penetration at approximately 100 nm spatial resolution, displacing 50–1000 times less cellular material and enabling sustained intracellular probing for over 30 min without disrupting the cytoskeleton or perturbing cellular calcium homeostasis. Additional capabilities of this nanoendoscope included mechanical flexibility, magnetic controllability (conferred by filling the nanotube walls or lumen with superparamagnetic Fe_3_O_4_ nanoparticles), gold nanoparticle functionalization of the tip for SERS.

**Figure 2:**
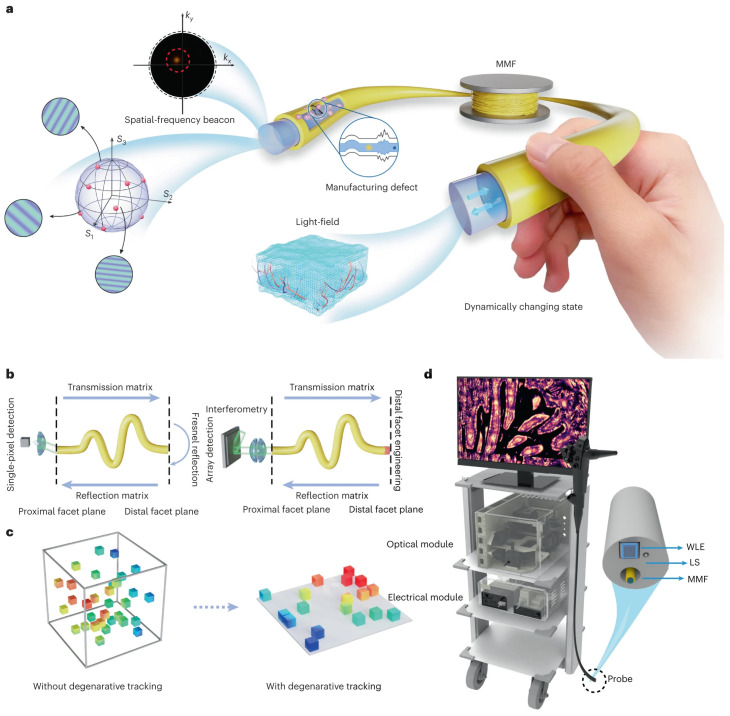
(**a**) STABLE detects and tracks MMF (multimode fiber) disorders from movements and manufacturing defects, enabling high-SNR imaging. Input light spans all spatial-frequency modes across the Poincaré sphere (S1–S3). The spatial-frequency beacon is located at the Fourier plane of the fiber proximal end (kx, ky). The star marks a localized manufacturing defect in the MMF core that distorts the transmitted wavefront. (**b**) Spatial-frequency beacon (**left**) vs. conventional speckle monitoring (**right**). In STABLE, the modulated wavefront reflects off the distal facet and refocuses to a single pixel in Fourier space; conventional monitoring uses array detection instead. (**c**) STABLE exploits MMF radial cylindrical symmetry to compress a high-order transmission problem into a much lower-order one. (**d**) Endoscope prototype (probe outlined in black). WLE, white-light endoscopy; LS, light source; MMF, multimode fiber. Reprinted from [56]. Copyright 2023. Springer Nature. Published under a Creative Commons Attribution 4.0 International (CC BY 4.0) license.

A significant advance in multimode fiber-based optical endoscopy was reported by Wen et al., who developed the spatial-frequency tracking adaptive beacon light-field-encoded (STABLE) endoscopy technique for in vivo imaging through a single thin multimode fiber with subdiffraction spatial resolution of 250 nm (Figure 2) [56]. The technique operates by co-propagating a reference optical beam, a spatial-frequency beacon, through the fiber alongside the imaging signal. Because this beacon always focuses to a single, predictable point in the Fourier plane at the fiber’s proximal end, any mechanical perturbation causes a measurable shift of that focal point, enabling real-time update of the transmission matrix at rates up to 1 kHz.

Full-vector light modulation with fluorescence emission difference detection further enhances the signal-to-noise ratio, achieving subdiffraction resolution of 250 nm. This design directly addresses a key limitation of multimode fibers: their extreme sensitivity to bending and mechanical perturbation, which degrades the transmitted wavefront and had previously prevented stable endoscopic imaging. Cross-scale imaging was demonstrated in a bronchus model, and in vivo subcellular imaging was validated in mouse models, establishing the feasibility of this approach for minimally invasive investigation of disease mechanisms in living biological systems. It should be noted that unlike the nanowire- and nanotube-based endoscopes described above, STABLE operates as a tissue-level imaging platform rather than an intracellular probe. Its relevance to this section lies in its achievement of subdiffraction spatial resolution through a minimally invasive single fiber, establishing an upper bound on optical performance achievable with fiber-based endoscopic systems.

The platforms discussed in this section illustrate how the field of optical nanoendoscopy has developed over the past two decades, moving from relatively simple tapered fiber tips toward increasingly complex designs that incorporate plasmonic enhancement, nanowire waveguiding, and computational wavefront correction. A comparison of the key performance parameters across the platforms reviewed in this section is provided in Table 1. In terms of tip dimensions, the probes span nearly three orders of magnitude: from sub-100 nm nanotube endoscopes and 100–250 nm SnO_2_ nanowire devices to the ~200 nm tapered fiber tips and, at the other extreme, the multimode fiber core of 105 μm used in STABLE. This dimensional range directly determines the degree of membrane invasiveness. while the STABLE system avoids intracellular insertion entirely. Regarding biocompatibility and probing duration, the MWCNT endoscope stands out as the most thoroughly characterized. Sustained intracellular probing for more than 30–40 min has been validated with no measurable perturbation of Ca^2+^ homeostasis, actin cytoskeleton, or mitochondrial function [54,55]. The STABLE system, operating as a non-penetrating tissue endoscope, demonstrated imaging stability over seven days ex vivo [56], a timescale not achievable by any intracellular probe. Functional versatility also differs markedly between platforms. The tapered fiber [42] and STABLE devices are primarily imaging tools, whereas the nanowire endoscope additionally enables quantitative pH sensing [43] and the MWCNT endoscope uniquely combines fluid transport (attolitre volumes), SERS-based molecular fingerprinting, electrochemical detection, and magnetic manipulation within a single probe body. Across all these architectures, it becomes clear that optical probes alone capture only one dimension of cellular activity. This observation motivates the integration of electrochemical detection into fiber-based probe designs.

## 3. Optical Electrodes

### 3.1. Fiber Probes with Integrated Optical and Electrochemical Channels

Optical electrodes serve not only as light guides but also as micro/nanoelectrodes for simultaneous collection of both electrochemical and photochemical responses with high resolution and selectivity [26]. These probes consist of three functional components: an optical fiber core serves as the waveguide, a conductive coating deposited on the fiber surface that functions as the working electrode, and an outer insulating layer. Optical fibers are typically made of doped silicon dioxide or electrically inert plastic materials. A conductive layer must be deposited on the fiber surface. Suitable conductor materials include gold, platinum and carbon [57]. Optical fiber electrodes are broadly classified into three geometries depending on the location of the conductive layer relative to the fiber tip. In disk electrodes the conductive coating is applied directly to the distal face of the fiber. In ring electrodes the conductive layer surrounds the lateral surface near the tip. In lateral surface electrodes, the coating extends along the fiber sidewall. Each geometry involves a distinct relationship between the optical aperture and the electroactive surface, and this spatial relationship determines the degree to which light transmission and electrochemical detection can be performed simultaneously and independently [58]. Conductive coatings are typically optically opaque, even when deposited as thin films. This creates a direct conflict between electrical conductivity and optical transparency. The problem is most acute in disk electrode configurations, where the conductive layer is deposited onto the same distal face that serves as the optical aperture. Any improvement in electrode sensitivity achieved by increasing film thickness comes at the direct expense of optical throughput, and vice versa. To overcome this limitation of light transmission through an electrically conductive surface ITO is typically chosen, which provides over 80% visible light transmission while maintaining sufficient electrical conductivity (resistivity of ~10^−4^ Ω cm) and can be deposited on the end of optical fibers using magnetron sputtering or sol–gel processes [59]. This section focuses on optical fibers bearing electrochemically active surface coatings.

Among the earliest demonstrations of this approach was a device enabling simultaneous chemical analysis and sample imaging using a single 350 μm diameter optical fiber containing approximately 6000 individual fibers with diameters of 3–4 μm [60]. The distal surface of the fiber was functionalized with a thin polymer layer (~2 μm) containing an immobilized fluorescent indicator or enzyme-indicating system. Two sensor types were developed: a pH sensor based on poly(HEMA) incorporating N-fluorescein acrylamide and an acetylcholine biosensor. In the latter, enzymatic hydrolysis of acetylcholine generates acetic acid, whose dissociated protons quench the fluorescence of the immobilized dye in proportion to substrate concentration (LOD 35 μM, response time <1 s, linear range 0.1–5.0 mM). A key advantage of this approach is the ability to alternately acquire visual and fluorescent information. When switching from filtered excitation light to unfiltered white light, the same CCD camera records an image of the object with a resolution of 4–4.4 μm, as demonstrated using mouse fibroblast imaging [60].

The concept of multimodal fiber sensors was further developed with the introduction of an electrochemically modulated fluorescence sensor based on an imaging fiber electrode. The sensor was fabricated by depositing a translucent gold film approximately 20 nm thick on the distal end of a fiber bundle, followed by modification with a thin Nafion film containing the immobilized cationic dye rhodamine B isothiocyanate (RBITC). This design combined quasi-reversible electrochemistry (preserving the characteristic redox peaks of RBITC and Ru(bpy)_3_^2+^) with optical transparency of approximately 30% in the visible range, enabling simultaneous fluorescence recording and control of the indicator’s redox state via the electrode potential. The hydrogen peroxide detection mechanism is based on H_2_O_2_ diffusing into the Nafion layer and irreversibly oxidizing the reduced form of RBITC, resulting in a decrease in fluorescence intensity by tens of percent even at submillimolar concentrations. A key advantage of the proposed architecture is the ability to spatially resolve the distribution of reactive oxygen species with electrochemical control of the sensor layer. This makes the device promising for studying the local production of H_2_O_2_ and other ROS in cell cultures and tissues, as well as for in situ monitoring of oxidative stress at micrometer spatial resolution [61].

The designs described above share a common limitation: the optical and electrochemical functions are carried by the same coated surface, which constrains the choice of materials and the achievable optical throughput. An alternative geometry that decouples these two functions at the probe tip was introduced by Pennarun et al. with the micro-optical ring electrode (MORE) [62]. Rather than coating the distal fiber face with a semitransparent conductor, MORE employs a thin-ring microelectrode geometry in which a commercial quartz optical fiber (radius 125 μm) serves as the insulating core, and a thermally deposited gold layer of ~600 nm thickness forms the conductive ring. This configuration delivers light directly to the electrochemical measurement region without intensity attenuation by a metal or semiconductor layer, allowing the use of moderate-power light sources (200 W Xe-Hg lamp). The photoelectrochemical capabilities of MORE were demonstrated using the phenothiazine dye methylene blue (MB^+^), for which direct electrochemical detection of the short-lived triplet state of ^3^MB^+^ was achieved for the first time. Under illumination, both photoanodic currents associated with the oxidation of ^3^MB^+^ to the radical cation MB^2+^ and photocathodic currents were recorded, with the reverse reduction of MB^2+^ inhibited by the inverse Marcus effect with a reorganization energy of ~0.3 eV. In the presence of the sacrificial electron donor Fe^2+^, an enhancement of the photoanodic response was observed due to the oxidation of photochemically generated leucomethylene blue with a lifetime of >3 s. A key advantage of MORE is its ability to detect photogenerated particles with lifetimes shorter than 90 µs [62].

A further development in this direction was the introduction of electro-optical hybrid microprobes, which integrate a microelectrode and an optical fiber within a single probe body for simultaneous measurement of extracellular ion fluxes and intracellular fluorescence signals. Smith and co-workers developed two configurations. In the potentiometric configuration, a single-mode optical fiber (125 µm diameter, 4 µm core, ~50 nm tip) is inserted through the housing of a calcium-selective microelectrode with a Fluka I ionophore and extends ~1 µm beyond the electrode opening (~2 µm). The amperometric configuration consists of a laser-pulled optical fiber with a sputtered gold coating, insulated with epoxy resin, forming a thin-ring microelectrode for oxygen detection. The optical channel delivers light from an argon laser (488 nm) to excite a fluorescent reporter (Calcium Green), pre-microinjected into the cell, followed by emission recording using a CCD camera or photomultiplier. The electrochemical channel operates in self-referencing mode: the probe oscillates between two positions (typically 10–30 μm apart) at 0.1–0.5 Hz, and the differential signal between the proximal and distal positions within the diffusion gradient is recorded. This approach effectively cancels baseline drift and background noise that are inherent to static microelectrode measurements [63].

In demonstration experiments on bag cell neurons of the mollusk *Aplysia*, cell depolarization with 60 mM KCl caused an increase in the intracellular Ca^2+^ concentration as measured by Calcium Green fluorescence and a synchronous increase in the outward Ca^2+^ flux measured by a calcium-selective electrode, which is interpreted as the activation of plasma calcium pumps that remove excess calcium after its entry through voltage-gated channels. In parallel, the authors developed a self-referencing enzyme biosensor for glucose detection, where glucose oxidase is immobilized on a platinum surface (diameter ~6 μm) and coated with cellulose acetate. H_2_O_2_ is oxidized at +600 mV (vs. Ag/AgCl), generating a current proportional to the glucose concentration. This allowed measurement of a glucose flux of 79 pmol cm^−2^ s^−1^ and a cellular consumption of 58 ± 7 fmol nL^−1^ s^−1^ in HIT pancreatic β-cells [63].

A persistent challenge in shared-surface optrode design is the generation of photoelectric artifacts when the electrode and the light-emitting aperture are in close proximity. Spagnolo et al. addressed this problem by exploiting the intrinsic photonic properties of metal-coated tapered optical fibers to develop integrated ‘fibertrodes’ [29]. In a tapered waveguide, the progressive reduction in the fiber radius along the propagation axis modifies the transverse wavevector of the guided modes, generating light emission at an angle of approximately 24° with respect to the taper axis. This angled emission redirects photons away from the electrode surface, which is positioned only 10 μm from the optical window, thereby eliminating direct illumination of the recording site without requiring post hoc signal correction. In vivo validation in the striatum of *Adora2a*-Cre Ai32 mice and the somatosensory cortex of Thy1-ChR2 mice demonstrated artefact-free optogenetic activation and simultaneous extracellular recording of local field potentials and single-unit action potentials, with spike sorting resolving up to three distinct units.

Thus, the advantages of electro-optical probes include multiparameter capability, high spatial and temporal localization of measurements, stable oxygen sensor sensitivity in physiological solutions, and artifact reduction due to the combination of modalities. Limitations include fabrication complexity, limitations in probe geometry and the need for careful optical and electrochemical calibration.

### 3.2. Electrochemical Probes with Light Delivery Through a Glass Nanopipette

The use of nanopipettes as light guides offers an elegant solution for fabricating probes that combine localized irradiation and electrochemical detection. The principle of exploiting the glass wall of a capillary as an optical waveguide was established in the context of chemical sensing well before its integration with electrochemical detection. Early capillary waveguide optrodes demonstrated that a glass capillary can simultaneously serve as a sample compartment, a sensing element, and an inhomogeneous optical waveguide, guiding light through the capillary wall via total internal reflection [64]. The capillary geometry allows for the natural integration of an optical channel and electrochemical sensors within a single housing. This architecture enables precise illumination control at the micro- and nanoscale, which is essential for studying photoactive systems with high spatial resolution. The nanopipette design is particularly valuable in scanning photoelectrochemical microscopy (SPECM), which requires simultaneous detection of photogenerated species and accurate positioning of the light source relative to the surface under investigation.

**Figure 3:**
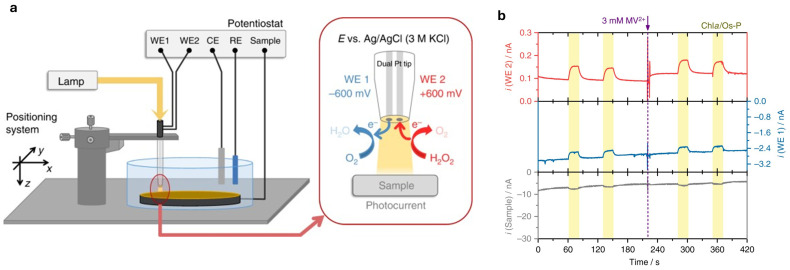
(**a**) SPECM investigation of the PS1/redox polymer-based photocathode. Schematic of the SPECM setup. Right: dual Pt microdisk tip enabling simultaneous detection of O_2_ and H_2_O_2_ (**b**) Generation of partially reduced oxygen species by inactivated PS1 and free Chla under illumination. Simultaneous photochronoamperometric responses for free Chla/Os-P electrodes. The sample and Pt microelectrodes were polarized at 0 mV, −600 mV (WE1), and +600 mV (WE2) vs. Ag/AgCl. Reprinted from [65]. Copyright 2018. Springer Nature. Published under a Creative Commons Attribution 4.0 International (CC BY 4.0) license.

The practical implementation of this approach is demonstrated in [65], where a double-barreled platinum microdisk electrode was created using a theta nanopipette and integrated into a SPECM system. The probe simultaneously serves as a localized light source and an electrochemical detector of O_2_ and H_2_O_2_ around a photosystem(PS) I biocathode (Figure 3). The glass shell of the capillary serves as a light guide, through which white light with an intensity of ~280 mW/cm^2^ is supplied to the area under the probe. One Pt disk is polarized at −600 mV (vs. Ag/AgCl) to record the diffusion-limited O_2_ reduction current, the second at +600 mV for amperometric H_2_O_2_ oxidation. Previously, Zhang et al. used an approach with structured mesoporous ITO electrodes for the direct adsorption of PS2 [66]. In contrast, a significantly more advanced methodology was applied in [65]. This dual-channel SPECM probing enables spatially and temporally resolved ROS monitoring, a key advantage over integral measurements, but requires precise setup positioning. The developed approach provided direct in situ detection of degradation products and a quantitative correlation between oxygen concentration and ROS generation, which would have been impossible with a standard three-electrode setup.

A further development was introduced by Yu et al., who replaced conventional Faradic current transduction with an iontronic photoelectrochemical approach [67]. A biomimetic light-driven ion pump was constructed by confining a PbS quantum dot/PEDOT:PSS photoelectric heterojunction within the asymmetric orifice of a borosilicate nanopipette. Upon 470 nm illumination at 130 mW cm^−2^, charge separation within the PbS QDs established a trans-pore potential gradient that drove unidirectional cation migration, generating a continuous photoinduced ionic current of approximately 135 pA at 0 V bias. The sensitivity of the photoinduced current to dissolved O_2_ arose from competitive capture of conduction-band electrons by O_2_, producing superoxide radical anions and thereby reducing the cation-driving charge gradient. This device achieved spatiotemporally resolved intracellular O_2_ measurements in single A549 cells across 20 subcellular positions within a single cell. Dynamic monitoring of mitochondrial respiration under FCCP and oligomycin treatment at mito-dense and mito-sparse subcellular regions confirmed the capability for chemotherapeutic evaluation at subcellular resolution.

The probes reviewed in Section 3.1 and Section 3.2 are summarized in Table 2. Within the shared-surface family, performance progresses from well-characterized optical biosensors [60,61] through ring electrode geometries capable of resolving sub-100 µs photogenerated transients [62], to self-referencing hybrid probes measuring femtomole-scale metabolic fluxes [63] and fibertrodes achieving sub-10 µV electrophysiological noise with fully suppressed photoelectric artifacts [29]. The nanopipette architectures of Section 3.2 shift the emphasis from analytical figures of merit toward spatial access: the dual Pt SPECM probe [65] enables spatially resolved simultaneous O_2_ and H_2_O_2_ detection.

Taken together, the two architectures described in this section represent complementary realizations of the same underlying principle: the capillary wall guides light to the measurement zone while the electrochemical function is housed within the lumen or at the tip, avoiding the optical throughput penalties that arise when a conductive coating is deposited directly on the light-guiding surface. Both approaches remain at an early stage of development, with demonstrations confined to a small number of analytes and cell types. Extending this architecture toward multiplexed detection, longer-term intracellular monitoring, and validation across diverse biological systems constitutes the principal direction for future work in this area.

## 4. Combined Optical and Electrochemical Probes with Separated Optical and Electrochemical Channels

The probe designs discussed in the preceding section share a common constraint. When the optical and electrochemical functions are assigned to the same surface or material layer, the choice of conductor affects optical transmission and vice versa. A distinct approach is to assign each modality to a physically separate channel within a single probe body, such that the optical waveguide, the electrochemical electrode, and a delivery channel operate independently. This separation of channels allows each component to be optimized on its own terms. The developed devices span a wide range of scales and complexity from sub-micrometer intracellular nanoprobes to flexible implantable fibers designed for chronic in vivo interfacing, and it is across this range that the most significant recent advances in separated-channel probe design have been made.

**Figure 4:**
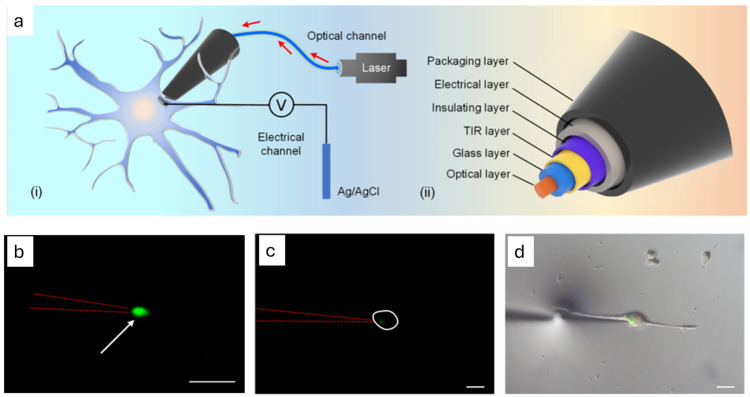
(**a**) Design of the opto-electrophysiology neural probe: (i) working schematic and (ii) explosion plot of each tip layer. (**b**) Fluorescence image of optical channel stimulation. The red dotted line shows the probe profile, and the arrow points to the stimulated cell. Scale bar: 100 μm. (**c**) Fluorescence image of intracellular optical stimulation. The red dotted line and the white solid line show the probe and cell profiles, respectively. Scale bar: 20 μm. (**d**) Merged image of intracellular optical stimulation. Scale bar: 20 μm. Reprinted with permission from [68]. Copyright 2024. American Chemical Society.

One recent example is an optoelectrophysiological probe incorporating an optical channel within the fiber core and an IrOx electrode on the outer surface, complemented by a total internal reflection (TIR) layer designed to prevent excitation light from reaching the conductive elements and thereby suppress photoelectric artifacts (Figure 4) [68]. The work addresses the challenge of improving the accuracy of opto-electrophysiological measurements at the level of single neurons by eliminating photoelectric artifacts, which are especially critical when recording weak intracellular currents in the voltage-clamp mode. IrOx electrodes were shown to have lower impedance at 1 kHz, higher capacitance and lower noise, while the shape of the tip was maintained after bending and repeated punctures of the agar phantom and cells. Using HT22 neurons, it was shown that a probe with a tip of ~1 μm penetrates into the cell with significantly less membrane damage than conventional tapered fibers, and intracellular and single-neuron optical stimulation is achieved by choosing the tip diameter and the configuration of one- or two-channel (473 and 665 nm) optical output (Figure 4). The contribution of this work is the demonstration of an intracellular optoelectrophysiological probe architecture that combines subcellular optical stimulation with near-complete elimination of photoelectric artifacts.

**Figure 5:**
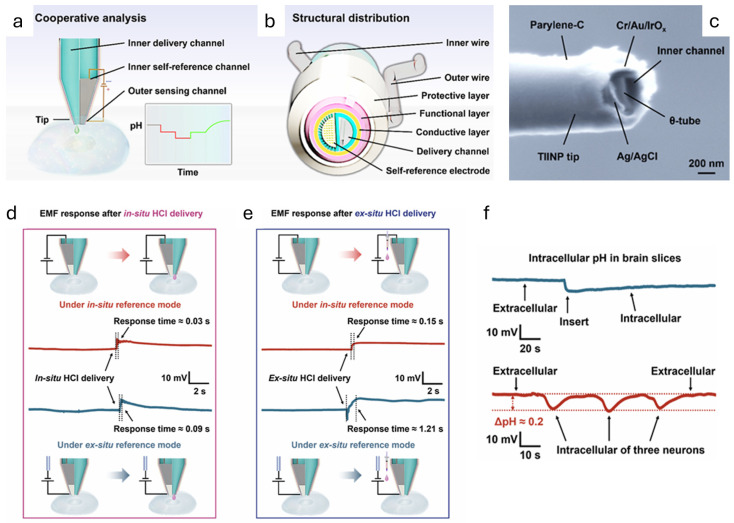
(**a**) Schematic diagram of real-time monitoring of intracellular pH variations during in situ delivery using TINP. (**b**) Structural distribution diagram of TINP. (**c**) SEM characterization of TINP tip. (**d**) EMF responses after in situ HCl delivery under different reference modes. (**e**) EMF responses after ex situ HCl delivery under different reference modes. (**f**) EMF response recorded during continuous insertion of TIINP into single neurons in brain slices. Reprinted with permission from [69]. Copyright 2026. American Chemical Society.

In a subsequent study the same authors shifted their focus from suppressing photoelectric artifacts in optoelectrophysiological measurements to expanding the functionality and reliability of the nanoelectrodes themselves, developing a triune intracellular nanoprobe (TINP) [69]. The proposed TINP is a theta-nanopipette with an IrOx-coated working electrode formed on the outer surface, one internal channel filled with Ag/AgCl and serving as an integrated self-reference electrode, and the second channel remaining free for local delivery (Figure 5a–c). Selective removal of the Parylene-C insulating nanolayer at the probe tip using an atmospheric plasma jet precisely defines the length of the exposed electroactive surface while preserving insulation along the probe body. This fabrication strategy simultaneously addresses two critical requirements. Firstly, it provides a controlled submicron operating window for intracellular pH and potential measurements with minimal membrane damage, and, secondly, it integrates in situ self-reference and local delivery, improving the accuracy of electromotive force (EMF) measurements and enabling the recording of transient and steady-state changes in intracellular pH induced by delivered agents at the level of single neurons and in brain slices.

Experiments on brain slices confirm the preservation of functionality of all three channels during intra-tissue implantation. However, the study highlights limitations related to the lack of long-term in vivo studies, the need for further miniaturization, and potential artifacts during simultaneous pH and electrical activity recording [69].

The two nanoprobe architectures described above both target the single-cell and single-neuron scale, where the primary challenges are minimizing membrane disruption, suppressing measurement artifacts, and maintaining metrological reliability in a highly confined intracellular environment. A different set of engineering priorities emerges when the target is not a single cell but a volume of neural tissue in a living, behaving animal. In this context, the dominant constraints shift toward long-term mechanical biocompatibility, scalable fabrication, and the integration of multiple modalities within a probe footprint that does not provoke a chronic inflammatory response. It is at this scale that the thermal drawing process introduced by Canales et al. offers its principal advantages.

Canales et al. presented a fundamentally new class of multifunctional neural probes fabricated using a thermal drawing process from polymer and polymer-metal composites [70]. The key novelty of this work lies in the integration of three modalities (optogenetic stimulation, electrophysiological recording, and microfluidic drug delivery) into a single flexible fiber device with mechanical properties comparable to those of neural tissue. The authors demonstrated for the first time the feasibility of long-term (up to 2 months) combined experiments in freely moving mice with simultaneous optical stimulation through embedded waveguides, recording of individual neuron activity, and local injection of pharmacological agents. A key achievement is the significant reduction in tissue reaction. Immunohistochemical analysis showed substantially reduced glial encapsulation (GFAP, Iba1, ED1) and blood–brain barrier disruption (IgG) compared to standard metal microwires. This opens prospects for developing stable brain-machine interfaces. Thermal drawing process technology enables the scalable production of thousands of identical probes from a single blank, dramatically reducing the cost of multimodal neurophysiological experiments.

The mechanical demands of spinal cord interfacing are more stringent than those of brain implantation: the spinal cord experiences tensile strains of up to approximately 12% during normal movement which conventional metallic electrodes cannot accommodate without developing cracks. Lu et al. addressed this by extending the thermally drawn fiber platform to the spinal cord, replacing conductive polymer composites with micrometer-thick meshes of silver nanowires (AgNWs) deposited onto the fiber surface by dip coating from isopropanol solution [71]. For applications requiring even greater deformability, a stretchable variant based on a cyclic olefin copolymer elastomer core sustained up to 200% tensile strain while preserving impedance within the range suitable for extracellular recording. The key advantage of the AgNW mesh geometry over continuous metallic films is its resilience to deformation: a three-layer mesh maintained low impedance at strains up to ~100%, which single-layer coatings could not achieve. In Thy1-ChR2-YFP transgenic mice, optical pulses delivered at 473 nm through the fiber core evoked spinal neural activity correlated with local field potentials recorded simultaneously by the concentric AgNW ring electrode, with EMG responses confirmed in the ipsilateral gastrocnemius muscle. Chronic implantation over one week in freely moving mice demonstrated stable recording noise levels and, importantly, only modest astrocytic response with no apparent disruption to surrounding neuronal populations.

The three probe architectures reviewed in this section share a common structural logic: by physically separating the optical, electrochemical, and delivery channels within a single probe body, each function can be independently optimized without compromising the others. This separation stands in deliberate contrast to the shared-surface designs discussed in previous section. At present, the most significant unresolved challenges are the validation of these probes under chronic in vivo conditions, the reduction in probe dimensions to the true nanoscale without sacrificing optical transmission or electrode sensitivity, and the extension of multimodal functionality beyond the brain tissue models in which most demonstrations have been conducted. Addressing these challenges will be essential for the transition from proof-of-concept neurophysiology tools to broadly applicable single-cell analytical platforms.

## 5. Opto-Electrodes for Scanning Probe Microscopy

**Figure 6:**
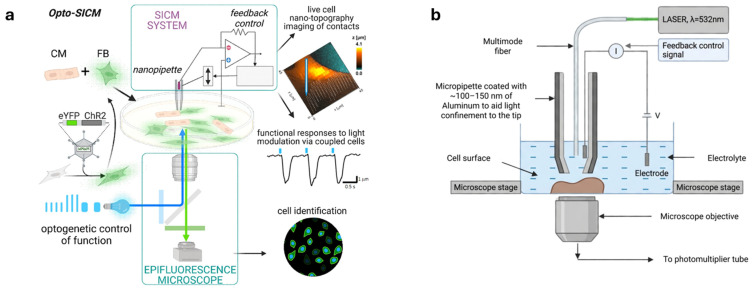
(**a**) Cardiac fibroblasts expressing ChR2-eYFP were co-cultured with unmodified cardiomyocytes. A controllable light source coupled to an inverted epifluorescence microscope provided selective optogenetic stimulation, while a hopping-mode SICM system simultaneously mapped fibroblast–cardiomyocyte topography and recorded cardiomyocyte contractions. Reprinted from [72]. Published under a Creative Commons Attribution 4.0 International (CC BY 4.0) license (**b**) Schematic diagram of the hybrid SICM-SNOM apparatus [73,74].

The development of scanning probe microscopy has driven the emergence of hybrid approaches that integrate electrochemical and optical imaging within a single instrument. In the earliest implementations, sample illumination was achieved using a standard epifluorescence unit without the use of optical fibers (Figure 6a) [72]. Currently there are two main approaches to combining scanning probe microscopy with optical methods. In the first, light is transmitted through a glass nanopipette and exits through its apical opening. In the second, the optical fiber is modified layer-by-layer to form a multifunctional ring probe, either by using dual-channel combined fiber electrodes or, in the case of SICM by embedding the optical fiber within a glass nanopipette (Figure 6b) [73,74]. The key element of such systems is a multifunctional probe capable of simultaneously recording the electrochemical response and the optical signal. The combination of SICM/SECM with optical microscopy allows for the simultaneous investigation of optical properties and the acquisition of electrochemical information, as well as photoelectrochemical studies of interfaces with high spatial resolution.

The first integration of SICM with near-field scanning optical microscopy (SNOM) was reported in [73]. The authors modified a standard SICM setup so that the ion current and the optical signal of scattered laser radiation (λ = 532 nm) were simultaneously recorded through a glass micropipette coated on the outside with aluminum. This configuration enabled concurrent topographic and near-field optical imaging of living rabbit cardiomyocytes in saline solution. A micropipette with an internal diameter of approximately 500 nm was positioned at a distance of approximately 250 nm from the cell surface, i.e., in the near field. The resulting maps demonstrated clearly distinguishable sarcomeric striations with a period of ~2.1 μm and a resolution of approximately 500 nm. Optical images complemented the SICM maps, providing more detailed visualization of Z-lines and Z-grooves [73].

The combination of scanning electrochemical microscopy with optical microscopy (SECM/OM) was pioneered by Lee and Bard who proposed an approach based on a gold-coated SNOM fiber completely insulated with an electrophoretic anodic varnish, forming a micrometer-scale ring electrode at the probe apex [75]. The resulting ring probes were shown to provide a stable steady-state current. The authors demonstrated that anodic electrophoretic coating of gold-coated SNOM probes enables simultaneous acquisition of micrometer-scale electrochemical and optical images of microstructures.

Spatial resolution in SECM/OM was substantially improved by Takahashi et al., who developed an approach based on SECM and optical microscopy using conical fiber-optic and glass capillary electrodes [76]. A combination of Ti/Pt-coated, parylene-insulated nanoprobes (effective electrode radius of approximately 35 nm, optical aperture <170 nm) coupled with quartz-tuned shear-force feedback enables precise topographic, electrochemical, and fluorescent imaging of objects with large height differences, including HRP-functionalized microspheres and living PC12 cells. Simultaneous recording of topography and oxygen current from a single living PC12 cell under physiological conditions was demonstrated for the first time, enabling local respiration rate assessment. Near-field fluorescence imaging with a resolution beyond the diffraction limit and triple (topography/current/optics) imaging of Au microelectrode structures are also demonstrated.

A highly reproducible method for fabricating nanometer-scale fiber-optic electrodes for combined SECM/OM was developed by Maruyama et al., based on selective chemical etching of GeO_2_-doped optical fibers in buffered HF solution, followed by gold sputtering and electrodeposition of an anodic electrophoretic varnish [77]. By controlling the tip shape (predominantly a pencil-like double taper with an angle of ~20°) and the number of insulation cycles, the authors obtained electrodes with radius from ≈100 to ≈5 nm, demonstrating stable sigmoidal voltammograms and diffusion-limited current. Compared with micrometer-sized electrodes, these probes provided substantially higher SECM/OM spatial resolution: electrochemical resolution of approximately 300 nm was achieved when imaging an implantable IDA electrode, while the optical channel was limited to approximately 930 nm owing to light leakage through the thin gold layer. The feasibility of recording the fine morphology of neurites in living PC12 cells in negative-feedback SECM mode was also demonstrated, highlighting the potential of nanometer-scale fiber-optic electrodes as multifunctional probes for high-resolution scanning of cellular microenvironments [77].

The first nanoscale photo-SECM with probe-side illumination was reported by Bae et al. [78]. The method is based on a glass-sealed metal nanoelectrode that simultaneously functions as an electrochemical sensor and a light guide, projecting a localized illumination spot onto the substrate. A key advantage of this configuration over lateral illumination geometries is the elimination of the shadowing effect caused by the probe body, which under lateral illumination produces slow transients. Platinum nanoelectrodes with a radius of 40–50 nm were used for photoelectrochemical mapping of ferrocene methanol oxidation and the oxygen evolution reaction on the surface of an Nb:TiO2 (110) single crystal. In the substrate generation/tip collection (SG/TC) mode, diffusion-controlled positive feedback was achieved at sufficient light power, and current transients at the tip were virtually lag-free. Spatial resolution in this approach is determined by the nanoelectrode radius, not the size of the illuminated region, which is critical for nanoscale studies of heterogeneous photoelectrochemical processes [78].

A significant practical limitation of early photo-SECM setups was the mechanical coupling between the rigid optical fiber and the piezoelectric positioner, which induced oscillations and positional discontinuities during nanoprobe scanning. Askarova et al. addressed this by implementing contactless optical delivery via a plano-convex lens system that collimates and focuses light from a 250 W HgXe lamp onto the back of the probe’s glass capillary, forming an approximately 1 mm diameter illumination spot with minimal intensity loss (from 6.5 to 5.8 mW) [79]. Platinum nanoelectrodes with radius of 80–170 nm were fabricated by drawing 25 μm diameter wire into borosilicate capillaries followed by polishing on a 50 nm aluminum oxide disk, with the glass shell serving as the light guide. Mechanical decoupling eliminated vertical oscillations with amplitudes of tens of nanometres at short tip-to-substrate distances—critical for accurate approach curves and probe protection. Validation on BiVO_4_ single crystals demonstrated artifact-free 3D surface images over 7 × 7 μm^2^ areas in negative-feedback mode, with complete consistency between forward and backward scan lines, representing a marked improvement over the mechanically coupled configuration. Control experiments on photooxidation of ferrocene methanol on Nb:TiO_2_ (0.5%) with a 60 nm radius probe at a tip-to-substrate distance of approximately 200 nm confirmed that illumination intensity and spatial resolution were preserved relative to the original technique.

The most recent contribution to this area is the development of MORE for SPECM. A gold-coated optical fiber (~155 μm outer diameter) is integrated into an epoxy-insulated glass capillary and polished to a planar geometry, yielding a probe that simultaneously functions as a light guide, a spectroscopic probe, and an electrochemical sensor. Electrochemical characterization in 1 mM FcCH_2_OH solution and numerical modeling in COMSOL Multiphysics Software confirmed reproducible ring electrode geometry and steady-state diffusion behavior. Integration into the SPECM system enabled simultaneous recording of UV–Vis–NIR optical spectra and electrochemical currents, with spectroelectrochemical experiments validating MORE’s capacity to monitor solution composition in the immediate vicinity of the electrode as a function of applied potential. For single cells of the alga (*Eremosphaera viridis*), linear and three-dimensional scans over 1000 × 1000 μm^2^ areas revealed a local increase in O_2_ reduction current (~0.5 nA) under fiber-optic illumination relative to dark conditions, confirming targeted microscale photostimulation with simultaneous electrochemical detection at the single-cell level [80].

The probes surveyed in this section trace a clear developmental trajectory: from the first proof-of-concept combinations of SICM and SNOM at the micrometer scale, through progressively smaller fiber-optic electrodes with radius reaching tens of nanometers, to fully integrated SPECM platforms capable of simultaneous topographic, electrochemical, and optical measurements at the single-cell level. Throughout this progression the central engineering challenge has remained consistent. At each successive generation the principal design objective was to bring the optical and electrochemical functions into close spatial register while preserving the performance of each channel. Table 3 summarizes the key parameters of these platforms and makes this evolution particularly visible in the optical channel. In the earliest hybrid instruments the optical function was essentially passive. The aluminum-coated micropipette of the SICM–SNOM system scattered laser light through its apical opening, providing near-field contrast but no spectroscopic selectivity and no control over the illuminated area. When gold-coated SNOM fibers were introduced as ring electrodes, the optical role shifted from passive detection to active local illumination, though the micrometer-scale aperture continued to limit spatial resolution. Reducing electrode radius to the sub-100 nm range changed this situation fundamentally. The glass sheath of the probe itself became the light guide, confining illumination to an area comparable in size to the electrochemical sensing zone and, in through-tip geometries, removing the shadowing artifacts that plague side-illumination configurations. The MORE platform takes this logic one step further by adding a full UV–Vis–NIR spectroscopic channel. The optical output is therefore no longer a spatial reference alone but a chemically resolved signal acquired simultaneously with the electrochemical current.

## 6. Conclusions, Challenges and Potentials

The work reviewed in the preceding sections reflects a sustained effort to combine two fundamentally different modes of cellular interrogation within a single miniaturized probe. Optical nanoendoscopes have demonstrated that subcellular structures can be visualized with nanometer-scale spatial resolution and minimal membrane perturbation. Opto-electrochemical probes with shared or separated channels have shown that light delivery and electrochemical detection can be co-registered at the same cellular location. Multifunctional fiber probes for neural interfaces have proven capable of simultaneous optogenetic actuation and electrophysiological recording in freely moving animals. SPECM platforms based on optical fiber electrodes have extended photoelectrochemical imaging to the single-cell level. Taken together, these architectures establish that the integration of optical and electrochemical modalities into a single fiber-based probe is not only feasible but can deliver analytical capabilities that neither modality achieves in isolation.

Several challenges have emerged as consistent barriers across all of the probe classes examined. Fabrication reproducibility remains a significant concern, since the manual assembly steps required by many hybrid probe designs introduce geometric variability that complicates quantitative comparison between experiments and limits throughput. The fundamental tension between optical transparency and electrical conductivity in shared-channel architectures has been only partially resolved by ITO and ring electrode geometries, and further progress in transparent conducting materials or photonic engineering will be necessary to extend these approaches to the nanometer scale. In separated-channel and neural probe designs, the mechanical mismatch between stiff probe bodies and soft neural or epithelial tissue continues to pose risks of chronic tissue damage and signal degradation, despite recent advances in elastomeric fiber drawing and nanowire mesh electrodes. Elastomeric fiber drawing enables the integration of stretchable conductors and optical waveguides into ultraflexible polymer fibers, while nanowire mesh electrodes form mechanically compliant, conformal networks that can follow tissue micromotions with minimal added stiffness [81,82]. For SPECM probes, the spatial resolution of the optical channel remains constrained by light leakage through metallic coatings, and the range of biologically validated model systems is still narrow.

The potential of these technologies is nonetheless considerable. The most immediate opportunity lies in extending validated probe architectures to multiplexed analyte detection. Most current designs report a single electrochemical parameter, whereas the biological questions that motivate this field frequently require simultaneous measurement of two or more analytes. Paired indicators such as O_2_ and H_2_O_2_ are needed to assess mitochondrial function, while pH and dopamine together report on synaptic activity. Advances in functionalization chemistry and multi-electrode fiber geometries suggest that this barrier is tractable. A second and more demanding challenge is the transition from in vitro validation to genuine in vivo deployment, where probe dimensions, biocompatibility, and long-term signal stability impose requirements that most current designs do not yet meet. Progress toward this goal will require not only continued refinement of individual probe architectures but also closer convergence between fabrication materials science, surface chemistry, and biological validation. The field is still at an early stage in this respect, and the distance between proof-of-concept demonstrations in isolated cell lines and reliable operation in complex living tissue remains substantial.

## Figures and Tables

**Table 1:** Comparative performance parameters of optical nanoendoscopes for single-cell imaging.

Probe Type	Tip Diameter	Spatial Resolution	Modality	Excitation Wavelength	Cell/Tissue Model	Cell Viability/Biocompatibility	Ref.
Chemically etched single-mode fiber	~200 nm (tip); 8 μm (base)	~100 nm (FDTD simulation)	Fluorescence	532 nm; broadband (external source)	Human fibroblasts; HepG2 cells; MDA-MB-231; *C. elegans* AM140 (in vivo)	No membrane damage; 3 viability assays passed; ≥90% safe penetrations	[42]
SnO_2_ nanowire on tapered fiber	100–250 nm (wire); 300–500 nm (fiber)	2 μm (two QD clusters resolved)	Fluorescence, QD delivery	442 nm 325 nm UV	HeLa	No cell death, apoptosis, significant cytosolic stress, or membrane rupture after nanowire insertion.	[43]
MWCNT on glass micropipette	50–200 nm OD; 50–60 μm length	~100 nm	Fluorescence, SERS, fluid transport	785 nm (SERS); 488 nm (fluorescence);	HeLa cells; human osteosarcoma cells; rat primary hepatocytes	No necrosis (>90% penetrations); ~50–1000× less displacement vs. glass; Ca^2+^ rise delayed/small/transient;	[54,55].
STABLE—multimode fiber	Single MMF; core 105 μm	250 nm (subdiffraction)	Fluorescence (light-field encoded), differential fluorescence microscopy (subdiffraction); volumetric 3D fluorescence	488 nm	Sheep small intestine ex vivo (5 days); pig esophagus; mouse gastrointestinal tract in vivo	Single thin fiber; ethics-approved in vivo; no cell-level toxicity data; stable focusing ≥1 week.	[56]

**Table 2:** Comparative performance parameters of fiber probes with integrated and separated opto-electrochemical channels.

Probe/Electrode Type	Probe Geometry	Electrode Material	Analyte(s)	Detection Potential/Mode	Light Source/Wavelength	Cell/Biological Model	Ref.
350 μm imaging fiber bundle + poly(HEMA) layer	Disk	Fluorescent indicator/enzyme	pH; Acetylcholine	Optical (fluorescence quenching by H^+^)	Broadband; blue excitation filter	Mouse fibroblasts	[60]
Imaging fiber bundle + Au semitransparent film + Nafion/RBITC layer	Disk	Au (~20 nm semitransparent film)	H_2_O_2_ (ROS)	Electrochemical oxidation; redox state controlled by applied potential	Visible (fluorescence excitation of RBITC)	Cell cultures and tissues (ROS/oxidative stress monitoring)	[61]
MORE: quartz fiber + thermally deposited Au ring	Ring	Au ring (~600 nm thick)	Triplet ^3^MB^+^; photogenerated species	Photoanodic/photocathodic; applied range −0.4 to +1.0 V vs. SCE	200 W Xe-Hg lamp	Solution/photoelectrochemical model system	[62]
Fibertrode: metal-coated tapered fiber	Tapered	Pt (~7–15 μm diam.)	LFP; action potentials	Extracellular recording; spike sorting up to 3 units;	473 nm (ChR2 optogenetic activation)	Mouse striatum (Adora2a-Cre Ai32); somatosensory cortex (Thy1-ChR2) in vivo	[29]
Electro-optical hybrid: Ca^2+^-selective microelectrode (Fluka I ionophore) + single-mode fiber	Coaxial	Pt (~6 μm., GOx + cellulose acetate for glucose); Ag/AgCl (ion-selective)	Ca^2+^ flux; O_2_; Glucose	Potentiometric (Ca^2+^); amperometric +600 mV vs. Ag/AgCl (H_2_O_2_/glucose)	488 nm (Ar laser)	Aplysia bag cell neurons; HIT pancreatic β-cells	[63]
Theta nanopipette: dual Pt microdisk electrodes;	Dual-disk	Pt (two independent disks)	O_2_; H_2_O_2_	−660 mV (O_2_ reduction); +600 mV (H_2_O_2_ oxidation) vs. Ag/AgCl	White light ~280 mW cm^−2^	PS I/redox polymer biocathode (photoelectrochemical model)	[65]
PbS QD/PEDOT:PSS heterojunction in asymmetric borosilicate nanopipette orifice (iontronic)	Nanopipette orifice	No metal electrode; photoinduced ionic current (PIC) transduction	O_2_ (intracellular)	Photoinduced ionic current ~135 pA at 0 V bias	470 nm	A549 single cells	[67]

**Table 3:** Comparison of opto-electrodes for scanning probe microscopy.

Probe/Technique	Electrode Radius	Optical Resolution/Aperture	Analyte	Biological Application	Tip-Sample Distance Control	Ref.
SICM + SNOM (Al-coated micropipette)	~250 nm	~500 nm	Ion current (topography)	Rabbit cardiomyocytes	Ion current (SICM) feedback; tip held ~250 nm from surface	[73]
SECM/OM—Au-coated SNOM fiber + varnish	Micrometer ring	Micrometer-scale	Electrochemical current	Microstructure imaging	SECM feedback current or shear force (tuning fork)	[75]
SECM/OM—Ti/Pt parylene nanoprobe	~35 nm	<170 nm aperture	O_2_ current	PC12 cells	Shear force (tuning fork, STA mode)	[76]
GeO_2_ fiber + Au sputtering + varnish	5–100 nm	~930 nm	Electrochemical current	PC12 neurites	Shear force (tuning fork) or SECM current; fixed-height mode for cell imaging	[77]
Contactless-delivery Pt nanoelectrode (SPECM)	60–170 nm	-	BiVO_4_ photocurrent	BiVO_4_ crystal surface	Approach curve; tip manually positioned; vibration-free contactless coupling	[79]
MORE in SPECM (Au-coated fiber in capillary)	Ring, ~155 μm OD	UV–Vis–NIR spectroscopy	O_2_ reduction	*Eremosphaera viridis* (single cell)	Constant-height mode; approach curve used for initial positioning	[80]

## Data Availability

No new data were created or analyzed in this study.

## Abbreviations

The following abbreviations are used in this manuscript:

AFM	Atomic force microscopy
AgNW	Silver nanowire
ChR2	Channelrhodopsin-2
EMF	Electromotive force
GFAB	Glial fibrillary acidic protein
IFE	Imaging fiber electrode
ITO	Indium tin oxide
LFP	Local field potential
MB	Methylene blue
MMF	Multimode fiber
MWCNT	Multiwalled carbon nanotube
MORE	Micro-optical ring electrode
PS	Photosystem
QDs	Quantum dots
RBITC	Rhodamine B isothiocyanate
ROS	Reactive oxygen species
RNS	Reactive nitrogen species
SECM	Scanning electrochemical microscopy
SICM	Scanning ion-conductance microscopy
SERS	Surface-enhanced Raman scattering
SNOM	Scanning near-field optical microscopy
SPECM	Scanning photoelectrochemical microscopy
STABLE	Spatial-frequency tracking adaptive beacon light-field-encoded
TIR	Total internal reflection
TINP	Triune intracellular nanoprobe
YFP	Yellow fluorescent protein

## References

[1] Marcuccio F., Chau C.C., Tanner G., Elpidorou M., Finetti M.A., Ajaib S., Taylor M., Lascelles C., Carr I., Macaulay I. (2024). Single-Cell Nanobiopsy Enables Multigenerational Longitudinal Transcriptomics of Cancer Cells. Sci. Adv..

[2] Xu C., Yang D., Wang Y., Liu R., Wang F., Tian Z., Hu K. (2024). Micro/Nanoelectrode-Based Electrochemical Methodology for Single Cell and Organelle Analysis. Nano Res..

[3] Actis P., Maalouf M.M., Kim H.J., Lohith A., Vilozny B., Seger R.A., Pourmand N. (2013). Compartmental Genomics in Living Cells Revealed by Single-Cell Nanobiopsy. ACS Nano.

[4] Lindström S., Andersson-Svahn H. (2011). Miniaturization of Biological Assays—Overview on Microwell Devices for Single-Cell Analyses. Biochim. Biophys. Acta Gen. Subj..

[5] Bulbul G., Chaves G., Olivier J., Ozel R., Pourmand N. (2018). Nanopipettes as Monitoring Probes for the Single Living Cell: State of the Art and Future Directions in Molecular Biology. Cells.

[6] Hu K., Nguyen T.D.K., Rabasco S., Oomen P.E., Ewing A.G. (2021). Chemical Analysis of Single Cells and Organelles. Anal. Chem..

[7] Zheng X.T., Li C.M. (2012). Single Cell Analysis at the Nanoscale. Chem. Soc. Rev..

[8] Winey M., Meehl J.B., O’Toole E.T., Giddings T.H. (2014). Conventional Transmission Electron Microscopy. Mol. Biol. Cell.

[9] Hickey S.M., Ung B., Bader C., Brooks R., Lazniewska J., Johnson I.R.D., Sorvina A., Logan J., Martini C., Moore C.R. (2021). Fluorescence Microscopy—An Outline of Hardware, Biological Handling, and Fluorophore Considerations. Cells.

[10] Yeh J.I., Shi H. (2010). Nanoelectrodes for Biological Measurements. Wiley Interdiscip. Rev. Nanomed. Nanobiotechnol..

[11] Clausmeyer J., Schuhmann W. (2016). Nanoelectrodes: Applications in Electrocatalysis, Single-Cell Analysis and High-Resolution Electrochemical Imaging. TrAC Trends Anal. Chem..

[12] Sciurti E., Biscaglia F., Prontera C.T., Giampetruzzi L., Blasi L., Francioso L. (2023). Nanoelectrodes for Intracellular and Intercellular Electrochemical Detection: Working Principles, Fabrication Techniques and Applications. J. Electroanal. Chem..

[13] Vaneev A.N., Timoshenko R.V., Gorelkin P.V., Klyachko N.L., Korchev Y.E., Erofeev A.S. (2022). Nano- and Microsensors for In Vivo Real-Time Electrochemical Analysis: Present and Future Perspectives. Nanomaterials.

[14] Timoshenko R.V., Vaneev A.N., Savin N.A., Klyachko N.L., Parkhomenko Y.N., Salikhov S.V., Majouga A.G., Gorelkin P.V., Erofeev A.S. (2020). Promising Approaches for Determination of Copper Ions in Biological Systems. Nanotechnol. Russ..

[15] Liu Y.-L., Zhao Y.-X., Li Y.-B., Ye Z.-Y., Zhang J.-J., Zhou Y., Gao T.-Y., Li F. (2022). Recent Advances of Nanoelectrodes for Single-Cell Electroanalysis: From Extracellular, Intercellular to Intracellular. J. Anal. Test..

[16] Zhang X., Huang K., Lai X., Liu X., Jiang H., Wang X. (2025). Applications and Prospects of Micro/Nanoelectrodes in Single-Cell Imaging and Electrochemical Detection. TrAC Trends Anal. Chem..

[17] Zhang C., Du X., Zhang Z. (2025). Development of Micro/Nanoelectrodes for Single-Cell Analysis. Sens. Actuators Rep..

[18] Lei L., Guo C., Yang H.B., Hu F. (2026). Recent Advances in Single-Cell Electrochemical Sensing Technology: A Review. Microchem. J..

[19] Vaneev A.N., Gorelkin P.V., Akasov R.A., Timoshenko R.V., Lopatukhina E.V., Garanina A.S., Abakumova T.O., Aleksandrin V.V., Salikhov S.V., Edwards C.R.W. (2024). In Vitro/In Vivo Oxygen Electrochemical Nanosensor for Bioanalysis. J. Electroanal. Chem..

[20] Timoshenko R.V., Gorelkin P.V., Vaneev A.N., Krasnovskaya O.O., Akasov R.A., Garanina A.S., Khochenkov D.A., Iakimova T.M., Klyachko N.L., Abakumova T.O. (2024). Electrochemical Nanopipette Sensor for In Vitro/In Vivo Detection of Cu^2+^ Ions. Anal. Chem..

[21] Zhang Y., Takahashi Y., Hong S.P., Liu F., Bednarska J., Goff P.S., Novak P., Shevchuk A., Gopal S., Barozzi I. (2019). High-Resolution Label-Free 3D Mapping of Extracellular PH of Single Living Cells. Nat. Commun..

[22] Liu C., Zhao Y., Cai X., Xie Y., Wang T., Cheng D., Li L., Li R., Deng Y., Ding H. (2020). A Wireless, Implantable Optoelectrochemical Probe for Optogenetic Stimulation and Dopamine Detection. Microsyst. Nanoeng..

[23] Xue H., Wang L., Yao H., Shen S., Zhao X., Yuan C., Yu L., Chen G., Liu J. (2025). Single-Cell Endoscopy for Multifunctional Live-Cell Molecular Analysis. Biosensors.

[24] Chisanga M., Masson J.-F. (2024). Machine Learning–Driven SERS Nanoendoscopy and Optophysiology. Annu. Rev. Anal. Chem..

[25] Teng P., Wen X., Liu Z., Zhang J., Zhang Y., Copner N., Yang J., Li K., Bowkett M., Gao D. (2022). An Optical Fiber Integrated Optoelectrode for the Photoelectrochemical Detection. Opt. Commun..

[26] Thomas N., Singh V., Kuss S. (2021). Optical Fibers in Analytical Electrochemistry: Recent Developments in Probe Design and Applications. TrAC Trends Anal. Chem..

[27] Jiang S., Song J., Zhang Y., Nie M., Kim J., Marcano A.L., Kadlec K., Mills W.A., Yan X., Liu H. (2021). Nano-Optoelectrodes Integrated with Flexible Multifunctional Fiber Probes by High-Throughput Scalable Fabrication. ACS Appl. Mater. Interfaces.

[28] Eynaki H., Kiani M.A., Golmohammadi H. (2020). Nanopaper-Based Screen-Printed Electrodes: A Hybrid Sensing Bioplatform for Dual Opto-Electrochemical Sensing Applications. Nanoscale.

[29] Spagnolo B., Balena A., Peixoto R.T., Pisanello M., Sileo L., Bianco M., Rizzo A., Pisano F., Qualtieri A., Lofrumento D.D. (2022). Tapered Fibertrodes for Optoelectrical Neural Interfacing in Small Brain Volumes with Reduced Artefacts. Nat. Mater..

[30] Flusberg B.A., Cocker E.D., Piyawattanametha W., Jung J.C., Cheung E.L.M., Schnitzer M.J. (2005). Fiber-Optic Fluorescence Imaging. Nat. Methods.

[31] Yu X., Zhang S., Olivo M., Li N. (2020). Micro- and Nano-Fiber Probes for Optical Sensing, Imaging, and Stimulation in Biomedical Applications. Photonics Res..

[32] Penedo M., Miyazawa K., Okano N., Furusho H., Ichikawa T., Alam M.S., Miyata K., Nakamura C., Fukuma T. (2021). Visualizing Intracellular Nanostructures of Living Cells by Nanoendoscopy-AFM. Sci. Adv..

[33] Orth A., Ploschner M., Wilson E.R., Maksymov I.S., Gibson B.C. (2019). Optical Fiber Bundles: Ultra-Slim Light Field Imaging Probes. Sci. Adv..

[34] Choi Y., Yoon C., Kim M., Yang T.D., Fang-Yen C., Dasari R.R., Lee K.J., Choi W. (2012). Scanner-Free and Wide-Field Endoscopic Imaging by Using a Single Multimode Optical Fiber. Phys. Rev. Lett..

[35] Vo-Dinh T., Kasili P., Wabuyele M. (2006). Nanoprobes and Nanobiosensors for Monitoring and Imaging Individual Living Cells. Nanomedicine.

[36] Vo-Dinh T., Kasili P. (2005). Fiber-Optic Nanosensors for Single-Cell Monitoring. Anal. Bioanal. Chem..

[37] Flusberg B.A., Jung J.C., Cocker E.D., Anderson E.P., Schnitzer M.J. (2005). In Vivo Brain Imaging Using a Portable 3.9 Gram Two-Photon Fluorescence Microendoscope. Opt. Lett..

[38] Bulina M.E., Chudakov D.M., Britanova O.V., Yanushevich Y.G., Staroverov D.B., Chepurnykh T.V., Merzlyak E.M., Shkrob M.A., Lukyanov S., Lukyanov K.A. (2006). A Genetically Encoded Photosensitizer. Nat. Biotechnol..

[39] Wu Y., Leng Y., Xi J., Li X. (2009). Scanning All-Fiber-Optic Endomicroscopy System for 3D Nonlinear Optical Imaging of Biological Tissues. Opt. Express.

[40] Andrásfalvy B.K., Galiñanes G.L., Huber D., Barbic M., Macklin J.J., Susumu K., Delehanty J.B., Huston A.L., Makara J.K., Medintz I.L. (2014). Quantum Dot–Based Multiphoton Fluorescent Pipettes for Targeted Neuronal Electrophysiology. Nat. Methods.

[41] Andrasfalvy B.K., Zemelman B.V., Tang J., Vaziri A. (2010). Two-Photon Single-Cell Optogenetic Control of Neuronal Activity by Sculpted Light. Proc. Natl. Acad. Sci. USA.

[42] Cheemalapati S.V., Winskas J., Wang H., Konnaiyan K., Zhdanov A., Roth A., Adapa S.R., Deonarine A., Noble M., Das T. (2016). Subcellular and In-Vivo Nano-Endoscopy. Sci. Rep..

[43] Yan R., Park J.H., Choi Y., Heo C.J., Yang S.M., Lee L.P., Yang P. (2012). Nanowire-Based Single-Cell Endoscopy. Nat. Nanotechnol..

[44] Actis P., Mak A.C., Pourmand N. (2010). Functionalized Nanopipettes: Toward Label-Free, Single Cell Biosensors. Bioanal. Rev..

[45] Nguyen T.D., Song M.S., Ly N.H., Lee S.Y., Joo S.W. (2019). Nanostars on Nanopipette Tips: A Raman Probe for Quantifying Oxygen Levels in Hypoxic Single Cells and Tumours. Angew. Chem. Int. Ed..

[46] Dubkov S., Overchenko A., Novikov D., Kolmogorov V., Volkova L., Gorelkin P., Erofeev A., Parkhomenko Y. (2023). Single-Cell Analysis with Silver-Coated Pipette by Combined SERS and SICM. Cells.

[47] Xu D., Liang B., Xu Y., Liu M. (2023). Recent Advances in Tip-Enhanced Raman Spectroscopy Probe Designs. Nano Res..

[48] Kolmogorov V., Erofeev A., Vaneev A., Gorbacheva L., Kolesov D., Klyachko N., Korchev Y., Gorelkin P. (2023). Scanning Ion-Conductance Microscopy for Studying Mechanical Properties of Neuronal Cells during Local Delivery of Glutamate. Cells.

[49] Takahashi Y., Zhou Y., Miyamoto T., Higashi H., Nakamichi N., Takeda Y., Kato Y., Korchev Y., Fukuma T. (2020). High-Speed SICM for the Visualization of Nanoscale Dynamic Structural Changes in Hippocampal Neurons. Anal. Chem..

[50] Kolmogorov V.S., Erofeev A.S., Barykin E.P., Timoshenko R.V., Lopatukhina E.V., Kozin S.A., Gorbacheva L.R., Salikhov S.V., Klyachko N.L., Mitkevich V.A. (2023). Scanning Ion-Conductance Microscopy for Studying β-Amyloid Aggregate Formation on Living Cell Surfaces. Anal. Chem..

[51] Vaneev A.N., Gorelkin P.V., Barykin E.P., Kolmogorov V.S., Timoshenko R.V., Mitkevich V.A., Petrushanko I.Y., Varshavskaya K.B., Salikhov S.V., Klyachko N.L. (2025). Impact of Antioxidants on Mechanical Properties and ROS Levels of Neuronal Cells Exposed to Β-Amyloid Peptide. ChemBioChem.

[52] Shambat G., Kothapalli S.R., Provine J., Sarmiento T., Harris J., Gambhir S.S., Vučković J. (2013). Single-Cell Photonic Nanocavity Probes. Nano Lett..

[53] Niu J.J., Schrlau M.G., Friedman G., Gogotsi Y. (2011). Carbon Nanotube-Tipped Endoscope for in Situ Intracellular Surface-Enhanced Raman Spectroscopy. Small.

[54] Singhal R., Orynbayeva Z., Kalyana Sundaram R.V., Niu J.J., Bhattacharyya S., Vitol E.A., Schrlau M.G., Papazoglou E.S., Friedman G., Gogotsi Y. (2011). Multifunctional Carbon-Nanotube Cellular Endoscopes. Nat. Nanotechnol..

[55] Orynbayeva Z., Singhal R., Vitol E.A., Schrlau M.G., Papazoglou E., Friedman G., Gogotsi Y. (2012). Physiological Validation of Cell Health upon Probing with Carbon Nanotube Endoscope and Its Benefit for Single-Cell Interrogation. Nanomedicine.

[56] Wen Z., Dong Z., Deng Q., Pang C., Kaminski C.F., Xu X., Yan H., Wang L., Liu S., Tang J. (2023). Single Multimode Fibre for in Vivo Light-Field-Encoded Endoscopic Imaging. Nat. Photonics.

[57] Xu Y., Lu P., Chen L., Bao X. (2017). Recent Developments in Micro-Structured Fiber Optic Sensors. Fibers.

[58] Janik M., Koba M., Śmietana M. (2024). Optical Fiber Chemo and Biosensors Operating in the Electrochemical Domain—A Review. TrAC Trends Anal. Chem..

[59] Deiss F., Sojic N., White D.J., Stoddart P.R. (2010). Nanostructured Optical Fibre Arrays for High-Density Biochemical Sensing and Remote Imaging. Anal. Bioanal. Chem..

[60] Bronk K.S., Michael K.L., Pantano P., Walt D.R. (1995). Combined Imaging and Chemical Sensing Using a Single Optical Imaging Fiber. Anal. Chem..

[61] Khan S.S., Jin E.S., Sojic N., Pantano P. (2000). A Fluorescence-Based Imaging-Fiber Electrode Chemical Sensor for Hydrogen Peroxide. Anal. Chim. Acta.

[62] Pennarun G.I., Boxall C., O’Hare D. (1996). Micro-Optical Ring Electrode: Development of a Novel Electrode for Photoelectrochemistry. Analyst.

[63] Smith P.J.S., Haydon P.G., Hengstenberg A., Jung S.-K. (2001). Analysis of Cellular Boundary Layers: Application of Electrochemical Microsensors. Electrochim. Acta.

[64] Wolfbeis O.S. (1996). Capillary Waveguide Sensors. TrAC Trends Anal. Chem..

[65] Zhao F., Hardt S., Hartmann V., Zhang H., Nowaczyk M.M., Rögner M., Plumeré N., Schuhmann W., Conzuelo F. (2018). Light-Induced Formation of Partially Reduced Oxygen Species Limits the Lifetime of Photosystem 1-Based Biocathodes. Nat. Commun..

[66] Zhang J.Z., Sokol K.P., Paul N., Romero E., van Grondelle R., Reisner E. (2016). Competing Charge Transfer Pathways at the Photosystem II–Electrode Interface. Nat. Chem. Biol..

[67] Dou Y., Wang B., Jin M., Yu Y., Zhou G., Shui L. (2017). A Review on Self-Assembly in Microfluidic Devices. J. Micromech. Microeng..

[68] Xu Q., Xi Y., Wang L., Du Z., Xu M., Ruan T., Cao J., Zheng K., Wang X., Yang B. (2024). An Opto-Electrophysiology Neural Probe with Photoelectric Artifact-Free for Advanced Single-Neuron Analysis. ACS Nano.

[69] Du Z., Xu Q., Xi Y., Yang X., Xu M., Peng Q., Zheng K., Wei N., Jiang C., Wang L. (2026). A Triune In Situ Integrated Nanoprobe with Controlled Exposed Tip for Reliable Single-Neuron Analysis. ACS Nano.

[70] Canales A., Jia X., Froriep U.P., Koppes R.A., Tringides C.M., Selvidge J., Lu C., Hou C., Wei L., Fink Y. (2015). Multifunctional Fibers for Simultaneous Optical, Electrical and Chemical Interrogation of Neural Circuits in Vivo. Nat. Biotechnol..

[71] Lu C., Park S., Richner T.J., Derry A., Brown I., Hou C., Rao S., Kang J., Moritz C.T., Fink Y. (2017). Flexible and Stretchable Nanowire-Coated Fibers for Optoelectronic Probing of Spinal Cord Circuits. Sci. Adv..

[72] Song Q., Alvarez-Laviada A., Schrup S.E., Reilly-O’Donnell B., Entcheva E., Gorelik J. (2023). Opto-SICM Framework Combines Optogenetics with Scanning Ion Conductance Microscopy for Probing Cell-to-Cell Contacts. Commun. Biol..

[73] Korchev Y.E., Raval M., Lab M.J., Gorelik J., Edwards C.R.W., Rayment T., Klenerman D. (2000). Hybrid Scanning Ion Conductance and Scanning Near-Field Optical Microscopy for the Study of Living Cells. Biophys. J..

[74] Shevchuk A.I., Gorelik J., Harding S.E., Lab M.J., Klenerman D., Korchev Y.E. (2001). Simultaneous Measurement of Ca^2+^ and Cellular Dynamics: Combined Scanning Ion Conductance and Optical Microscopy to Study Contracting Cardiac Myocytes. Biophys. J..

[75] Lee Y., Bard A.J. (2002). Fabrication and Characterization of Probes for Combined Scanning Electrochemical/Optical Microscopy Experiments. Anal. Chem..

[76] Takahashi Y., Hirano Y., Yasukawa T., Shiku H., Yamada H., Matsue T. (2006). Topographic, Electrochemical, and Optical Images Captured Using Standing Approach Mode Scanning Electrochemical/Optical Microscopy. Langmuir.

[77] Maruyama K., Ohkawa H., Ogawa S., Ueda A., Niwa O., Suzuki K. (2006). Fabrication and Characterization of a Nanometer-Sized Optical Fiber Electrode Based on Selective Chemical Etching for Scanning Electrochemical/Optical Microscopy. Anal. Chem..

[78] Bae J.H., Nepomnyashchii A.B., Wang X., Potapenko D.V., Mirkin M.V. (2019). Photo-Scanning Electrochemical Microscopy on the Nanoscale with Through-Tip Illumination. Anal. Chem..

[79] Askarova G., Hesari M., Wang C., Mirkin M.V. (2022). Decoupling Through-Tip Illumination from Scanning in Nanoscale Photo-SECM. Anal. Chem..

[80] Thomas N., Singh V., Ahmed N., Trinh D., Kuss S. (2022). Single-Cell Scanning Photoelectrochemical Microscopy Using Micro-Optical-Ring Electrodes. Biosens. Bioelectron..

[81] Liu F., Fu T.-M. (2025). Material and Design Strategies for Chronically-Implantable Neural Probes. NPG Asia Mater..

[82] Wang S., Jiang Q., Liu H., Yu C., Li P., Pan G., Xu K., Xiao R., Hao Y., Wang C. (2024). Mechanically Adaptive and Deployable Intracortical Probes Enable Long-Term Neural Electrophysiological Recordings. Proc. Natl. Acad. Sci. USA.

